# Organophosphate Urinary Metabolite Levels during Pregnancy and after Delivery in Women Living in an Agricultural Community

**DOI:** 10.1289/ehp.7894

**Published:** 2005-07-18

**Authors:** Asa Bradman, Brenda Eskenazi, Dana B. Barr, Roberto Bravo, Rosemary Castorina, Jonathan Chevrier, Katherine Kogut, Martha E. Harnly, Thomas E. McKone

**Affiliations:** 1 Center for Children’s Environmental Health Research, School of Public Health, University of California, Berkeley, California, USA; 2 National Center for Environmental Health, Centers for Disease Control and Prevention, Atlanta, Georgia, USA; 3 Environmental Health Investigations Branch, California Department of Health Services, Oakland, California, USA; 4 Lawrence Berkeley National Laboratory and University of California, Berkeley, California, USA

**Keywords:** exposure, organophosphate, pesticides, pregnancy, prenatal, urinary metabolites, women

## Abstract

Little information has been published about pesticide exposures experienced by pregnant women. We measured six dialkyl phosphate (DAP) urinary metabolites of organophosphate (OP) pesticides in 600 pregnant, low-income women living in the Salinas Valley, California, an agricultural area. A total of 28% were employed as farm fieldworkers during pregnancy, and 81% had at least one household member who worked in agriculture. Samples were collected twice during pregnancy (mean = 13 and 26 weeks’ gestation, respectively) and just after delivery (mean = 9 days). As in other studies, dimethyldithiophosphate levels were higher than those of other urinary OP metabolites. Total DAP metabolite levels in samples collected after delivery were higher than in samples collected during pregnancy. Median metabolite levels at the first and second prenatal sampling points and at the postpartum collection were 102.8, 106.8, and 227.2 nmol/L, respectively. Both prenatal and postpartum metabolite levels were higher in these Salinas Valley women than in a sample of women of childbearing age in the general U.S. population (National Health and Nutrition Examination Survey), although the deviation from U.S. reference levels was most pronounced after delivery. Higher DAP metabolite levels in the immediate postpartum period may have implications for estimating dose during pregnancy and for exposure during lactation.

Approximately 340 million kilograms of agricultural pesticide active ingredient is used annually in the United States (Donaldson et al. 2002), and 85% of U.S. households store at least one pesticide for home use (Adgate et al. 2000; Whitmore et al. 1992). In 1993, the National Resource Council raised concerns that high levels of environmental pesticide exposure could compromise the health of U.S. children (National Research Council 1993). The Food Quality Protection Act of 1996, to address these concerns, mandates that the U.S. Environmental Protection Agency limit the amount and type of pesticides on food to levels deemed safe for children. In response to this legislation, several studies have measured the extent of pesticide exposure among the general public. Recent biologic monitoring studies indicate that pesticide exposures are widespread in the U.S. population, including children [Adgate et al. 2001; Barr et al. 2004; Centers for Disease Control and Prevention (CDC) 2001; Curl et al. 2003; Fenske et al. 2000; Koch et al. 2002; Loewenherz et al. 1997; Lu et al. 2000; O’Rourke et al. 2000; Shalat et al. 2003].

Few studies to date have focused specifically on exposure of children *in utero*. Those that have, however, indicate that pregnant women in the United States experience frequent exposures to pesticides (Berkowitz et al. 2003; CDC 2004; Eskenazi et al. 2004; Perera et al. 2003; Whyatt and Barr 2001; Whyatt et al. 2003). In a sample of 386 pregnant New York City women, Berkowitz et al. (2003) reported detectable urinary metabolites for pyrethroids, pentachlorophenol, and chlorpyrifos in 95, 94, and 80% of study participants, respectively. Whyatt et al. (2003) and Perera et al. (2003) have detected diazinon and chlorpyrifos in the air and dust of New York City homes and in the blood samples of pregnant women residing within them. Finally, studies have found metabolites for organophosphates (OPs), pentachlorophenol, naphthalene, *ortho*-phenylphenol, and several other pesticides in amniotic fluid (Bradman et al. 2003) and infant meconium (Whyatt and Barr 2001). Overall, these studies indicate that detectable pesticide exposures are occurring among pregnant women and their fetuses.

In the present study, we report OP metabolite levels in urine samples collected during and just after pregnancy from a low-income, primarily Latina cohort of women residing in an agricultural region of California.

## Materials and Methods

### Study location characteristics.

The Center for the Health Assessment of Mothers and Children of Salinas (CHAMACOS) is a community/university partnership investigating environmental allergen, pesticide, and other toxicant exposures experienced by women and children in Salinas Valley, California, an agricultural area. In 2001, approximately 240,000 kg of OP pesticide active ingredient were applied in this area, a level typical of recent years (California Department of Pesticide Regulation 2001). Of these pesticides, 42% were dimethyl OP pesticides, 38% were diethyl OP pesticides, and 20% did not devolve into a dialkyl phosphate (DAP) metabolite. In addition, approximately 5% of study participants reported home use of OP pesticides, although > 40% used other classes of pesticides in the home (Bradman A, unpublished data).

### Study population.

Between September 1999 and November 2000, 601 pregnant women were enrolled in the CHAMACOS birth cohort study. Women were contacted at six local prenatal clinics. Women were eligible to participate in this study if they were ≤20 weeks’ gestation at the time of enrollment, were ≥18 years of age, were qualified to receive poverty-based government health insurance, and planned to continue receiving prenatal care at a participating health center. Written informed consent was obtained from all participants in accordance with procedures approved by the University of California Berkeley Committee for the Protection of Human Subjects. Detailed descriptions of the Salinas Valley study area and the CHAMACOS study population have been published previously (Eskenazi et al. 2003).

### Interviews.

Participants’ demographic, health, and household information was collected through personal interviews. Interviews were conducted in English or Spanish by bilingual, bicultural study staff. A baseline interview occurred shortly after enrollment in the study, generally at 13 weeks’ gestation [mean (± SD) = 13.4 ± 5.2 weeks’ gestation]. Follow-up interviews occurred at approximately 26 weeks’ gestation (mean ± SD = 25.9 ± 2.6 weeks’ gestation) and after delivery (mean ± SD = 8.8 ± 17.9 days). Urine samples were collected at each interview.

Gestational age at urine collection was calculated for most women using the clinical estimate of gestational age at birth noted in the medical record. For women who miscarried or dropped from the study before delivery, gestational age was calculated using the reported date of last menstrual period and, when possible, verified by ultrasound (Eskenazi et al. 2004).

### Urine collection and analysis.

Spot urine samples were collected according to the procedures outlined by the CDC for use in the National Health and Nutrition Examination Survey (NHANES) 1999–2000 (CDC 2003). Women voided into a sterile urine cup in bathroom facilities at our field office or in the CHAMACOS mobile clinic. Specimens were aliquoted into precleaned glass containers with Teflon-lined caps, bar coded, and stored at –80°C until shipment. Samples were shipped on dry ice to the CDC and stored at –70°C until analysis.

### Pesticide and creatinine measurement.

We measured six nonspecific urinary OP metabolites, including three dimethyl phosphates, dimethylphosphate (DMP), dimethyldithiophosphate (DMDTP), and dimethylthiophosphate (DMTP); and three diethyl phosphates, diethylphosphate (DEP), diethyldithiophosphate (DEDTP), and diethylthiophosphate (DETP). Urine specimens were codistilled with acetonitrile. The DAP metabolites were derivatized to their chloropropyl esters. The concentrated extracts were then analyzed by isotope dilution gas chromatography–tandem mass spectrometry (Bravo et al. 2002), which is widely regarded as the definitive technique for trace analysis with DAP metabolite detection limits of ≤1 ppb (Barr et al. 1999; Shealy et al. 1996). Creatinine concentrations in urine were determined using a commercially available diagnostic enzyme method (Vitros CREA slides, Ortho Clinical Diagnostics, Raritan, NJ).

Laboratory quality control (QC) included repeat analysis of two in-house urine pools enriched with known amounts of pesticide residues whose target values and confidence limits were previously determined (Westgard 2003). Detection limits ranged from 0.05 μg/L for DEDTP to 1.2 μg/L for DMP. A total of 135 laboratory and 121 (blind) field QC samples were analyzed, representing 16% of total samples. Mean relative recoveries for six metabolites in laboratory QC samples ranged from 98 to 105% [coefficients of variation (CVs) ranged from 11 to 15%]. Average recovery of total DAP metabolites in field spikes ranged from 92 to 103% (CVs ranged from 4 to 9%). The mean level of DAP metabolites in 32 blank field samples was < 1 μg/L.

We assigned an imputed value of the limit of detection (LOD)/√2 to levels below the detection limit (Barr et al. 1999; Hornung and Reed 1990). Because many OP pesticides devolve to more than one metabolite in their class (diethyl or dimethyl phosphates), quantities were converted to molar concentrations (nanomoles per liter) and summed to obtain the total concentrations of the diethyl and dimethyl phosphates.

The creatinine concentration in each urine sample was reported in milligrams of creatinine per deciliter of urine. One sample with missing creatinine concentration data and three urinary creatinine levels that implied unreasonably high fluid consumption rates (< 10 mg/dL) were excluded from statistical analyses.

Of the 601 women who enrolled in the study, adequate urine samples with credible creatinine levels were collected from 590 (98%) women at the first prenatal sampling point, 498 (83%) women at the second prenatal sampling point, and 489 (81%) women after delivery.

### Data analysis.

The primary objective of this analysis was to present descriptive information about urinary OP metabolite levels during and soon after pregnancy. Geometric means and percentiles for individual and total dimethyl phosphate metabolites, individual and total diethyl phosphate metabolites, and total DAP metabolites were calculated for each sampling time point.

Total DAP metabolite concentrations were log-normally distributed, whereas the dimethyl and diethyl phosphate molar concentrations were not. Thus, we used the Spearman’s rank test to assess the correlation of metabolites (untransformed) between the different sampling points. We used general estimating equations (STATA GEE population-averaged model; StataCorp, College Station, TX) to determine the within- and between-individual variability for the 485 women with two measurements during pregnancy. We used paired *t*-tests and binary tests of proportions to compare each prenatal urinary metabolite measurement with the postpartum measurement. In addition, we calculated each participant’s ratio of DEP to DETP + DEDTP and DMP to DMTP + DMDTP metabolites at the three urine collection time points and then calculated the mean and median of these ratios. Finally, we used the Kolgomorov-Smirnov test of equality of distributions to compare the pregnancy and postpartum metabolite levels. We also used the Kolgomorov-Smirnov test and quantile regression to compare levels for the CHAMACOS cohort with levels measured at the same CDC laboratory for women participating in NHANES, a cross-sectional study of the U.S. population (CDC 2004). The 1999–2000 NHANES sample included 96 pregnant women and 271 nonpregnant women between 18 and 40 years of age (Barr et al. 2004; CDC 2004). We applied no sample weights to the NHANES data.

For statistical analyses, we present results that are not adjusted for creatinine levels. All analyses were repeated with creatinine-adjusted values to confirm our results. Analyses were conducted using STATA software (version 8.2; StataCorp).

## Results

### Demographic characteristics.

Eighty-five percent of CHAMACOS study participants were born in Mexico, with 48% having spent < 5 years in the United States. The mean age of participating women was 26 years, and nearly all lived within 200% of the federal poverty level. Twenty-eight percent of women were employed as farm workers at some point during their pregnancy, and 81% percent shared a home with at least one agricultural worker during their pregnancy. Additional demographic information on this population is presented in Eskenazi et al. (2004).

### Creatinine.

Creatinine levels consistently decreased from the first prenatal sample through postpartum, with median levels of 98.3 mg/dL [interquartile range (IQR) = 51.6–139.3], 90.6 mg/dL (IQR = 60.8–130.7), and 85.2 mg/dL (IQR = 51.6–122.4) at the first prenatal, second prenatal, and postpartum sampling times, respectively. This trend is consistent with medical reference data that indicate lower creatinine excretion in later trimesters and early postpartum (Becker et al. 1992; Davison et al. 1980; Davison and Noble 1981).

### Urinary metabolite concentration data.

Tables 1 and 2 present the unadjusted and the creatinine-adjusted geometric means, percentiles, and ranges for the six DAP metabolites and total diethyl and dimethyl phosphate molar concentrations at each of the three sampling points. Postpartum urinary metabolite levels were consistently higher than the prenatal samples, with median unadjusted total DAP metabolite levels of 102.8 nmol/L (IQR = 37.7–277.5), 106.8 nmol/L (IQR = 58.1–223.9), and 227.2 nmol/L (IQR = 96.0–554.6) and median creatinine-adjusted total DAP metabolite levels of 112.7 nmol/L (IQR = 56.3–316.0), 126.4 nmol/L (IQR = 68.8–237.8), and 283.5 nmol/L (IQR = 109.8–730.3) at the first prenatal, second prenatal, and postpartum sampling times, respectively. Detection frequencies for dimethyl, diethyl, and total DAP metabolites were higher at the second prenatal and postpartum sampling points than at the first prenatal sampling point. Dimethyl phosphate metabolite levels were higher than diethyl metabolites, a finding consistent with previous study results in other populations (Barr et al. 2004; CDC 2001 CDC 2003; Fenske et al. 2000).

**Figure 1:**
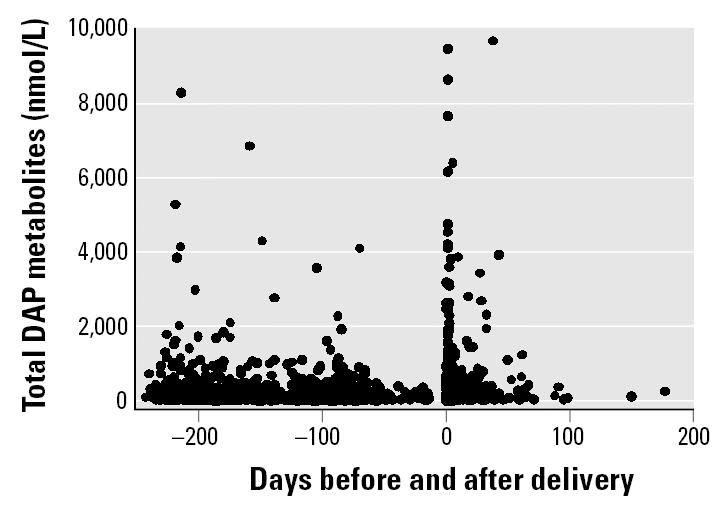
Total DAP urinary metabolite levels by days before and after delivery (*n* = 535 CHAMACOS women). The *y*-axis is truncated at 10,000 nmol/L, excluding five postpartum samples with higher DAP measurements. Prenatal data are shown for pregnancies with a known delivery date; 67 samples from 61 women who miscarried or dropped from the study before delivery are excluded from this graph. Delivery = day 0.

As presented in Tables 1 and 2, postpartum diethyl, dimethyl, and DAP metabolite levels were higher across the entire distribution compared with the prenatal sampling points. Figure 1 presents a scatter plot of the pregnancy and postpartum total urinary DAP metabolite levels by days before and after delivery. Metabolite levels vary widely both before and after birth, although there is greater variability immediately after birth. Paired *t*-tests for total DAP levels found significant mean differences between prenatal and postpartum measures. The mean difference in metabolite levels between the first and second prenatal sample was 109.0 nmol/L (*p* = 0.98), but the mean difference between the first prenatal and postpartum samples was 423.4 nmol/L (*p* < 0.001), and between the second prenatal and postpartum sampling points was 566.2 nmol/L (*p* < 0.001). The within-individual variability across sampling points was about twice the between-individual variability (SD = 1.09 and 0.40, respectively).

The proportions of women with urinary metabolite levels increasing between the first and second prenatal samples, first prenatal sample and postpartum, and second prenatal sample and postpartum were 53, 65, and 66%, respectively. Thus, there was an approximately even chance of either decreasing or increasing metabolite levels during pregnancy, whereas a significantly higher proportion of women had higher levels after delivery compared with prenatal levels (binary test of proportions *p* < 0.001). Identical trends were found within each season, indicating that the pattern is pregnancy related and not a temporal trend (data not shown). Creatinine adjustment enhanced the difference between prenatal and postpartum levels [mean difference = 791.3 and 970.4 nmol/g (*p* < 0.001) for the first prenatal vs. postpartum samples and second prenatal vs. postpartum samples, respectively].

We found a clear upward shift in the ratio of the diethyl phosphate metabolite DEP compared with the diethyl thiophosphate metabolites (DETP, DEDTP) between the prenatal and postpartum samples. The average ratios were 2.5 (SD = 6.0), 1.2 (SD = 3.5), and 8.8 (SD = 20.7) for the prenatal 1, prenatal 2, and postpartum samples, respectively. Similarly, the median ratios were 0.9, 0.2, and 3.2 for the prenatal 1, prenatal 2, and postpartum samples, respectively. The ratios of DMP to DMTP + DMDTP, however, remained relatively constant across the sampling time points. The median DMP:(DMTP + DMDTP) ratios were 0.3, 0.4, and 0.3 for the prenatal 1, prenatal 2, and postpartum samples, respectively.

Spearman correlations between the three sampling time points for the diethyl phosphate metabolites ranged from 0.03 to 0.07 (*p* = 0.4), for dimethyl phosphate metabolites ranged from 0.05 to 0.10 (*p* < 0.05–0.3), and for the total DAP metabolites ranged from 0.04 to 0.13 (*p* < 0.01–0.3). Overall, the correlation analyses indicated little or no correlation between the different sampling times. Within each sampling time cross-section (prenatal sample 1, prenatal sample 2, and postpartum), the correlations of dimethyl and diethyl phosphate metabolites were 0.43 (*p* < 0.01), 0.30 (*p* < 0.01), and 0.29 (*p* < 0.01), respectively. This finding suggests that some participants were exposed simultaneously to dimethyl and diethyl OP pesticides.

### Comparison with NHANES data.

**Figure 2:**
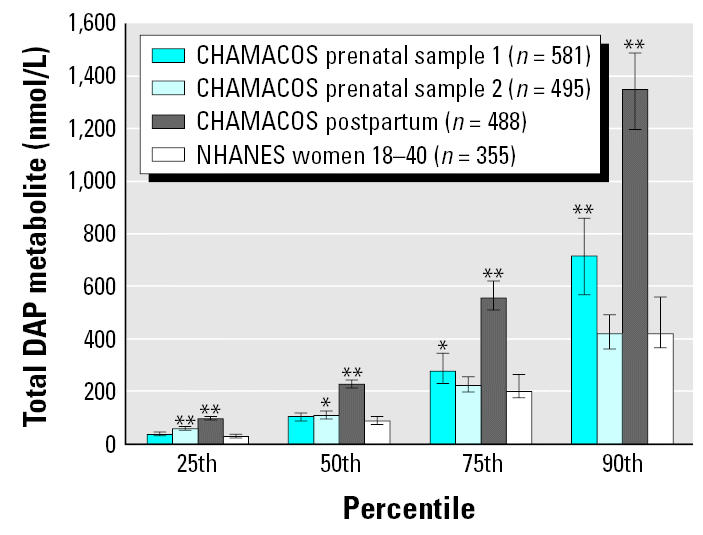
Total DAP urinary metabolite levels in the CHAMACOS cohort and NHANES 1999–2000. Error bars indicate 95% confidence intervals. Statistically significant differences between CHAMACOS sample and NHANES sample: *p < 0.05; **p < 0.001.

Tables 3 and 4 present unadjusted and creatinine-adjusted geometric means, percentiles, and ranges for the total diethyl and dimethyl phosphate molar concentrations among pregnant and nonpregnant women from the NHANES population. Within the NHANES sample, DAP metabolite levels were not significantly different between pregnant and nonpregnant women or between Mexican-American and non-Hispanic women (data not shown) (Barr et al. 2004). Thus, Figure 2 shows data for CHAMACOS women and all women between 18 and 40 years of age in the NHANES sample.

The distribution of total DAP metabolite levels for CHAMACOS women’s first and second prenatal visits was significantly higher than NHANES levels (Kolgomorov-Smirnov *D* = 0.16, *p* < 0.001, and *D* = 0.18, *p* < 0.001, respectively). When we further examined the distribution using quantile regression, we found that, for the first prenatal samples, CHAMACOS total DAP metabolite levels were higher than NHANES levels at the 75th and 90th percentiles (*p* < 0.04 and *p* = 0.001, respectively); for the second prenatal samples, however, CHAMACOS total DAP metabolite levels were higher than NHANES levels at the 25th and 50th percentiles (*p* < 0.001 and *p* < 0.05, respectively) (Figure 2). The distributions of dimethyl and diethyl metabolite levels for CHAMACOS women were significantly higher than the NHANES levels in the first (dimethyl: *D* = 0.24, *p* < 0.001; diethyl: *D* = 0.28, *p* < 0.001) and second (dimethyl: *D* = 0.19, *p* < 0.001; diethyl: *D* = 0.24, *p* < 0.001) prenatal visits. Again using quantile regression, we found that dimethyl metabolite levels were consistently higher across all quartiles at the first prenatal visit (*p* ≤ 0.05 at 25th, 50th, and 75th percentiles; *p* < 0.01 at 90th percentile), but only significantly higher for the 25th and 50th percentiles at the second prenatal visit (*p* < 0.001 and *p* < 0.01, respectively). Conversely, diethyl metabolite levels were significantly higher than NHANES values at only the 25th percentile for the first prenatal visit (*p* < 0.001), but were significantly higher at all quartiles for the second prenatal visit (*p* ≤ 0.001 at 25th, 50th, 75th, and 90th percentiles).

In the postpartum period, total DAP, dimethyl phosphate, and diethyl phosphate metabolite levels from the CHAMACOS women were higher than levels for NHANES women 18–40 years of age across the distribution (*D* = 0.31, *p* < 0.001; *D* = 0.30, *p* < 0.001; and *D* = 0.26, *p* < 0.01 for total DAP, dimethyl, and diethyl metabolites, respectively). Using quantile regression, we found that total DAP, dimethyl phosphate, and diethyl phosphate metabolite levels were significantly higher than NHANES levels at every quartile (*p* < 0.001 at 25th, 50th, 75th, and 90th percentiles for DAPs, dimethyl and diethyl phosphate metabolites, respectively).

Creatinine levels in CHAMACOS pregnancy samples were no different than in NHANES pregnancy samples. However, non-pregnant NHANES women had significantly higher creatinine levels than did pregnant NHANES women, which is consistent with known biologic changes that occur during pregnancy (Becker et al. 1992; Davison and Noble 1981; Davison et al. 1980). CHAMA-COS participants’ creatinine-adjusted total dimethyl and diethyl phosphate and DAP metabolite levels were significantly higher than creatinine-adjusted levels for NHANES women 18–40 years of age (data not shown). In fact, the difference between the creatinine-adjusted DAP metabolite levels for the two populations was larger than the difference we found for unadjusted levels (data not shown).

## Discussion

In this initial study of serial DAP metabolite levels in pregnant and early postpartum women, we detected measurable levels of DAP metabolites in nearly all urine samples collected from low-income women in the agricultural region of the Salinas Valley, California. Levels in this population were substantially higher than for the U.S. women of comparable age who participated in the NHANES 1999–2000 study. We have noted in our serial sampling that, although median metabolite levels in urine collected at approximately 13 and 26 weeks’ gestation were similar, postpartum metabolite levels were about double the pregnancy levels. In addition, we found a clear upward shift in the ratio of the diethyl phosphate metabolite DEP compared with the thiophosphate metabolites (DETP + DEDTP) between the women’s prenatal and postpartum samples. Because DEP is a known breakdown product of the bioactivated oxon form of diethyl OP pesticides (e.g., chlorpyrifos-oxon, diazinon-oxon, etc.), this shift in metabolite ratios may indicate pregnancy-related changes in hepatic cytochrome P450 metabolism (Needham 2005). However, the ratios of the dimethyl OP pesticides remained relatively constant across the sampling time points. Thus, we have no clear explanation for this finding. Creatinine adjustment accentuated the difference between prenatal and postpartum metabolite levels in the postpartum period. Women in this largely agricultural cohort had median postpartum urinary DAP metabolite levels that were 2.5 times higher than those for NHANES women.

We cannot readily explain the apparent increase in OP metabolite levels and the upward shift in the ratio of DEP to DETP + DEDTP in the postpartum period. One possible explanation is that the physiologic changes that occur during pregnancy increase the body’s capacity to store OP pesticides and/or their metabolites, but that these excess stores are excreted soon after delivery. During pregnancy, women retain approximately 4–6 L fluid, gain approximately 3.4 kg fat, and increase their blood volume by 40–45% (Cunningham et al. 1997); these changes may represent new compartments where OP pesticides or metabolites could be stored until parturition. Conversely, urinary frequency and glomerular filtration increase during pregnancy (Becker et al. 1992; Cunningham et al. 1997; Davison et al. 1980; Davison and Noble 1981), suggesting that metabolite excretion may occur more efficiently in the prenatal period than postpartum. In addition, pregnancy-related changes in participants’ diet probably occurred over the course of this study. However, because it is unlikely that the women began eating more fruits and vegetables contaminated with pesticide residues in the peripartum period, it is not known how such dietary factors could explain the observed changes in DAP metabolite levels postpartum. Finally, we found that, in the CHAMACOS population, pregnant and postpartum women’s urinary creatinine levels were lower than those of the nonpregnant women in NHANES. This is consistent with known biologic changes that occur during pregnancy (Becker et al. 1992; Davison and Noble 1981; Davison et al. 1980) and again demonstrates the many metabolic differences between pregnant and nonpregnant women. Further research is needed to determine which physiologic and dietary changes, if any, affect the excretion of OP metabolites. In the absence of this information, it is unclear whether prenatal or postpartum metabolite levels more accurately reflect exposures to OP pesticides during pregnancy.

As has been reported in previous studies (Barr et al. 2004; CDC 2001 CDC 2003; Fenske et al. 2002), CHAMACOS participants’ dimethyl metabolite levels consistently exceeded diethyl levels, with DMTP predominating. The molar ratio of dimethyl to diethyl metabolites in participants’ urine is 9:1, which is higher than would be expected given the 3:2 ratio of dimethyl to diethyl OP pesticides that the California Department of Pesticide Regulation reports are used in the Salinas Valley (California Department of Pesticide Regulation 2001). This discrepancy may be explained by the longer environmental half-lives of dimethyl than diethyl OP pesticides or by alternate exposure pathways, such as diet and home pesticide use, which we have not explored here. Regardless of the exposure pathway, the significant correlations we observed between dimethyl and diethyl phosphate metabolites within each sampling time point (Spearman *r* = 0.29–0.43) suggest that some participants may experience concurrent exposures to dimethyl and diethyl OP pesticides.

This study has several limitations. We have treated urinary DAP metabolite levels as an indicator of exposure to OP pesticides. Recent research suggests, however, that urinary metabolites may reflect not only an individual’s contact with pesticide parent compounds, but also contact with metabolites present in the environment (Lu et al. 2005). Lu et al. (2005) have recently reported that DAP metabolites are present in fresh fruit juices as a result of OP pesticide degradation. It is not currently known whether exposure to DAP metabolites would result in the intact excretion of these compounds. Findings from one animal study suggest that exposure to diethyl phosphate metabolites results predominately in the excretion of inorganic phosphate (Imaizumi et al. 1993). Nonetheless, DAP metabolite levels may overestimate a woman’s exposure to OP pesticides and, in fact, reflect her exposure, in part, to metabolites already present in her environment.

Another limitation of this study is that we have relied on metabolite levels from single spot urine samples collected at different times of the day to characterize participants’ exposure. Kissel et al. (2005) have reported that same-day spot samples collected from children vary in metabolite concentration, but that the first morning void tends to reflect the day’s total metabolite excretion better than do other spot samples (Kissel et al. 2005). The lack of correlation between CHAMACOS participants’ metabolite levels at different time points may be due, at least in part, to this sampling scheme. The high intraindividual variability we observed suggests that additional spot samples, a same-time sampling scheme (e.g., first morning voids for each woman at each time point), or perhaps even 24-hr samples might better characterize women’s cumulative exposure to OP pesticides during pregnancy.

Further, we assigned an imputed value of the LOD/√2 to levels below the detection limit. This method is identical to procedures adopted by the CDC and frequently used in exposure assessment (Barr et al. 1999, 2004; CDC 2003; Hornung and Reed 1990). However, results may differ depending on how LODs are considered across studies and if LODs differ in comparison populations. Further exploration is needed to determine the appropriate method of comparing large data sets with different LODs.

In summary, we found that pregnant women living in an agricultural area had higher urinary metabolite levels of OP pesticides compared with the general U.S. population. Our finding of higher levels in the immediate postpartum period may have implications for estimating dose during pregnancy and for infant exposure from breast-feeding. Our future analyses will explore possible determinants of exposure—such as fieldwork and proximity to agricultural fields—that may explain the high urinary OP metabolite levels among women in this agricultural community relative to other U.S. women. In addition, we will attempt to clarify whether documented physiologic changes among these women (e.g., prenatal weight gain) influenced the degree to which their prenatal and postpartum metabolite levels differed.

## Figures and Tables

**Table 1:** OP urinary metabolite levels at three time points, during pregnancy, and postpartum (nmol/L).^a^

				Percentile		
Sample	Detection frequency (%)	Geometric mean	Range	50th	75th	90th
Prenatal sample 1 (*n* = 590)						
DMP	50.3	14.6	3.3–1190.5	6.7	37.3	104.8
DMTP	65.6	30.0	0.9–7042.3	28.9	119.7	331.0
DMDTP	48.6	9.6	0.4–26582.3	4.5	25.9	136.7
Total DM	80.2	82.9	4.8–34362.6	74.2	232.7	648.0
DEP	60.4	7.7	0.9–1039.0	5.3	16.9	46.2
DETP	49.1	4.4	0.4–417.6	2.5	7.6	24.1
DEDTP	45.6	1.4	0.4–236.6	1.1	2.0	4.8
Total DE	74.3	16.7	1.7–1319.3	14.1	32.2	70.9
Total DAP (*n* = 581)	88.5	112.6	8.2–34438.1	102.8	277.5	731.6
Prenatal sample 2 (*n* = 498)						
DMP	71.5	13.6	3.4–498.7	12.0	35.0	75.3
DMTP	97.6	37.3	1.0–2417.7	42.4	93.3	230.0
DMDTP	57.6	4.1	0.4–1456.0	6.6	20.3	72.6
Total DM	99.6	76.4	4.8–4057.6	76.3	156.3	338.6
DEP	39.8	3.3	0.9–585.3	0.9	17.2	43.9
DETP	98.6	11.5	0.4–408.4	12.5	29.1	50.4
DEDTP	12.3	0.5	0.4–93.6	0.4	0.4	2.3
Total DE	98.8	20.7	1.7–735.1	22.6	44.1	91.6
Total DAP (*n* = 495)	100.0	113.3	7.1–4098.3	106.8	223.9	421.7
Postpartum (*n* = 489)						
DMP	67.1	27.7	3.4–21855.5	29.3	86.2	293.2
DMTP	85.9	60.0	1.0–20576.7	70.6	225.3	721.5
DMDTP	56.9	3.9	0.4–1329.1	4.0	15.8	61.6
Total DM	93.7	169.9	4.8–21857.3	162.4	444.9	1321.4
DEP	81.4	14.4	0.9–658.1	17.5	41.3	91.4
DETP	65.9	3.6	0.4–595.2	3.5	10.6	23.2
DEDTP	29.2	0.7	0.4–18.2	0.4	1.1	2.8
Total DE	92.2	25.0	1.7–665.6	25.7	58.4	128.1
Total DAP (*n* = 488)	97.1	229.5	6.5–21866.6	227.2	554.6	1348.6

Abbreviations: DE, diethyl metabolites; DM, dimethyl metabolites.

a Detection limits from multiple batches of urinary metabolite data: DMP = 0.6–1.2 μg/L; DMTP = 0.2–1.1 μg/L; DMDTP = 0.08–1.0 μg/L; DEP = 0.2–0.8 μg/L; DETP = 0.09–0.6 μg/L; DEDTP = 0.05–0.3 μg/L. Values below detection limit = LOD/√2, consistent with NHANES data published by CDC (2003).

**Table 2:** Creatinine-adjusted OP urinary metabolite levels at three time points during pregnancy and postpartum (nmol/g).^a^

				Percentile		
Sample	Detection frequency (%)	Geometric mean	Range	50th	75th	90th
Prenatal sample 1 (*n* = 589)						
DMP	50.2	17.3	1.2–3958.3	13.4	41.5	156.7
DMTP	65.6	35.5	0.7–7027.0	33.7	123.0	398.3
DMDTP	48.6	11.3	0.1–24298.2	9.5	28.3	137.1
Total DM	80.2	98.2	3.3–31410.1	85.1	258.7	728.4
DEP	60.4	9.2	0.4–1749.1	8.2	22.1	47.8
DETP	49.1	5.3	0.2–1131.8	4.7	9.5	25.5
DEDTP	45.6	1.6	0.1–242.8	1.4	3.1	6.3
Total DE	74.3	19.8	0.8–2221.0	18.1	36.4	83.5
Total DAP (*n* = 580)	88.5	133.4	7.0–31479.1	112.7	316.0	792.3
Prenatal sample 2 (*n* = 498)						
DMP	71.5	15.8	1.4–710.9	15.2	40.4	89.8
DMTP	97.6	43.2	0.3–2609.1	45.5	109.9	237.8
DMDTP	57.6	4.7	0.1–862.1	6.3	23.3	69.8
Total DM	99.6	88.3	2.8–3485.8	82.0	182.1	424.7
DEP	39.8	3.8	0.3–488.5	1.9	18.8	45.3
DETP	98.6	13.3	0.4–472.4	15.0	32.4	65.8
DEDTP	12.3	0.6	0.1–82.3	0.5	0.8	2.1
Total DE	98.8	23.9	1.0–775.3	25.8	51.6	108.5
Total DAP (*n* = 495)	100.0	130.9	8.8–3724.8	126.4	237.8	478.6
Postpartum (*n* = 489)						
DMP	67.1	35.2	1.1–86109.2	30.5	130.5	429.2
DMTP	85.9	76.3	0.3–34011.1	93.3	322.0	1099.6
DMDTP	56.9	4.9	0.1–1653.1	5.5	20.0	78.0
Total DM	93.7	216.0	2.5–93692.5	213.0	654.6	1796.7
DEP	81.3	18.3	0.4–795.3	22.4	52.9	115.2
DETP	65.9	4.6	0.2–1608.6	5.2	14.3	32.0
DEDTP	29.2	0.9	0.1–95.2	0.8	1.7	4.0
Total DE	92.2	31.8	1.0–1612.1	34.1	77.2	144.2
Total DAP (*n* = 488)	97.1	292.2	5.2–93798.6	283.5	730.3	1936.3

Abbreviations: DE, diethyl metabolites; DM, dimethyl metabolites.

a Detection limits from multiple batches of urinary metabolite data are given in Table 1. Values below detection limit = LOD/√2, consistent with NHANES data published by CDC (2003).

**Table 3:** Urinary DAP concentrations (nmol/L) among pregnant women (*n* = 96) and nonpregnant women of childbearing age (*n* = 271) in NHANES 1999–2000.

				Percentile		
	Detection frequency (%)	Geometric mean	Range	50th	75th	90th
Pregnant women^a^						
Total DM	82.5	48.1	4.5–2606.6	50.0	195.7	421.1
Total DE	75.3	8.2	1.5–296.1	8.9	20.3	42.8
Total DAP	92.8	70.5	6.0–2610.5	72.0	246.2	437.7
Nonpregnant women of childbearing age^b^						
Total DM	82.6	52.0	4.5–19721.9	54.8	159.4	378.5
Total DE	76.8	11.6	1.5–1157.1	13.7	34.0	56.7
Total DAP	90.9	82.3	2.3–19724.1	90.0	201.0	417.6

Abbreviations: DE, diethyl metabolites; DM, dimethyl metabolites.

a The distributions of OP metabolite levels in pregnant and nonpregnant women of childbearing age in the NHANES study were not statistically different (see “Data analysis”). Pregnant women ranged in age from 15 to 50 years.

b Total DAP *n* = 270 because of missing DMTP data.

**Table 4:** Creatinine-adjusted urinary DAP concentrations (nmol/g creatinine) among pregnant women (*n* = 96) and nonpregnant women of childbearing age (*n* = 271) in NHANES 1999–2000.

				Percentile		
	Detection frequency (%)	Geometric mean	Range	50th	75th	90th
Pregnant women^a^						
Total DM	82.5	49.8	1.5–3727.3	50.7	168.2	408.4
Total DE	75.3	8.5	0.7–308.5	8.4	28.4	55.8
Total DAP	92.8	73.0	3.1–3783.0	75.2	213.8	435.8
Nonpregnant women of childbearing age^b^						
Total DM	82.6	41.3	1.4–16713.5	44.8	125.2	313.3
Total DE	76.8	9.3	0.4–2492.8	9.2	23.0	55.8
Total DAP	90.9	65.5	2.3–16715.3	67.1	155.9	370.3

Abbreviations: DE, diethyl metabolites; DM, dimethyl metabolites.

a The distributions of OP metabolite levels in pregnant and nonpregnant women of childbearing age in the 1999–2000 NHANES study were not statistically different (see “Data analysis”). Pregnant women ranged in age from 15 to 50 years.

b Total DAP *n* = 270 because of missing DMTP data.
